# A modulation format recognition and optical signal-to-noise ratio monitoring scheme based on residual network and Taylor score pruning

**DOI:** 10.1371/journal.pone.0333936

**Published:** 2025-10-13

**Authors:** Jinrong Liang, Yong Bao

**Affiliations:** 1 School of Information Engineering, Nanning University, Nanning, Guangxi Zhuang Autonomous Region, China; 2 School of Computer, Electronics and Information, Guangxi University, Nanning, Guangxi Zhuang Autonomous Region, China; Najran University College of Computer Science and Information Systems, SAUDI ARABIA

## Abstract

Investigating practical methods for real-time monitoring of modulation formats (MF) and optical signal-to-noise ratio (OSNR) in coherent optical communication systems is critical for advancing future dynamic and heterogeneous optical networks. In this work, we propose a residual network with an attention mechanism(SA-ResNet) to perform joint monitoring of MF and OSNR for mainstream quadrature phase shift keying (QPSK) and M-ary quadrature amplitude modulation (MQAM) signals, including 8QAM, 16QAM, 32QAM, 64QAM, and 128QAM. After applying Taylor pruning to the model, its floating-point operations (FLOPs) were reduced from 40.5 M to 9.5 M, and its parameter memory was decreased from 2.6 M to 0.5 M. Notably, following fine-tuning, the model still achieved 100% MF recognition accuracy and an average absolute error of 0.34 dB for OSNR estimation under a sample length of 16,000 and fiber length of 160 km. When the model is evaluated using 5-fold cross-validation, the average MF recognition accuracy is 99.988%, and the mean of average absolute errors for OSNR estimation is 0.32 dB. These results indicate that the proposed model has acceptable monitoring performance and requires relatively low computational resources, which makes it attractive for lightweight application scenarios of optical fiber monitoring systems.

## 1. Introduction

With the rise of various data services, such as Internet of Things, cloud computing, big data, and artificial intelligence, network traffic exhibits exponential growth [1]. To meet these demands, optical fiber communication networks will develop toward dynamic and heterogeneous architectures in the future [2]. Specifically, ‘dynamic’ describes networks where status, resource allocation, and service bearing are adjusted in real time based on time, demand, or external conditions, rather than remaining fixed. ‘Heterogeneous’ denotes networks composed of components with diverse technologies, protocols, devices, or architectures. Therefore, real-time and accurate optical performance monitoring is essential to adapt to the dynamic resource scheduling and heterogeneous component collaboration of such networks. In optical performance monitoring tasks, since the transmission performance of optical fibers mainly depends on the OSNR, and the carrier recovery module in the coherent optical receiver must be compatible with the received MF, the monitoring of MF and OSNR is critical to maintaining efficient network operation [3].

## 2. Related work

Methods for optical performance monitoring can be categorized into conventional approaches and artificial intelligence-based methods. The conventional optical performance monitoring methods for measuring OSNR, which are based on statistical moments [4,5], training sequences [6,7], and delay interferometry [8,9], each exhibit distinct characteristics. The statistical moment method leverages second- and fourth-order statistical moments for OSNR monitoring, offering insensitivity to frequency offsets and phase noise but suffering from performance limitations imposed by the employed equalizer. The training sequence method’s performance relies on the design and properties of training sequences, yet modifying the transmitter to insert sequences may degrade spectral efficiency. The delay interferometry-based method, which exploits signal light coherence, features simplicity, cost-effectiveness, ease of maintenance, and robustness against dispersion and differential group delay, but requires additional hardware deployment.

In terms of MF recognition, the method using normalized power distribution for classification is unaffected by frequency offsets, but distinguishing between M-ary phase-shift keying signals with similar power distributions remains a challenge [10]. Methods based on compressed sensing and higher-order cyclic cumulants have also been applied to classify M-ary phase-shift keying and M-ary quadrature amplitude modulation signals. Nevertheless, the computation of higher-order cyclic cumulants and the reconstruction process of compressed sensing involve high computational complexity, which may present obstacles for scenarios with stringent real-time requirements [11].

The above methods can realize OSNR estimation and MF identification, but often cannot realize simultaneous measurement of the two parameters (OSNR and MF). With the progress of artificial intelligence technology, machine learning has been applied to optical performance monitoring, including back-propagation artificial neural networks [12,13], principal component analysis [14], k-nearest neighbor [15], and support vector machines [16]. However, these traditional machine learning methods lack the ability to extract and share features in optical performance monitoring tasks [17]. Therefore, deep learning methods with automatic feature extraction capabilities have gained increasing attention from researchers in optical performance monitoring tasks.

Some researchers have proposed using deep learning to automatically extract features such as constellation diagrams [3,18], eye diagrams [19,20], spectrograms [21], and asynchronous delay tap sampling graphs [22], thereby achieving accurate MF recognition and OSNR estimation. In these deep learning-based studies, the MF recognition accuracy usually reaches 100%, and the OSNR estimation error is controlled within a certain range. These research results are based on multi-task learning, and all adopt user-defined models: Reference [3] used a multi-task binary convolutional neural network, References [18,20] used residual multi-task learning networks, and References [19,21,22] used multi-task convolutional neural networks. In addition, classic convolutional neural networks such as AlexNet and VGG [23,24] have also been applied. However, few of these deep learning-based studies have explored model compression techniques to further lightweight network models and reduce computational complexity—a critical requirement for practical deployment in resource-constrained optical monitoring systems.

Taylor pruning is computationally efficient, causes minimal accuracy loss to the model after pruning, can effectively reduce computational costs, and exhibits cross-layer adaptability [25]. For the self-attention mechanism, the representation of a single sequence is computed by associating information across different positions within the sequence; it has been applied to image classification and regression tasks, obtaining acceptable prediction results [26,27]. We propose to combine the self-attention mechanism with a residual network and apply the Taylor pruning method to filter pruning for model lightweighting, so as to reduce the model’s FLOPs and the size of its parameter memory.

## 3. Principle

### 3.1 Multi task learning

Owing to the ability of multi-task learning to share feature information among related tasks for joint task optimization and to improve model performance, we adopt multi-task learning models for MF recognition and OSNR estimation of optical signals. MF recognition is a classification task, and its loss function is the cross entropy loss function *loss*_*MF*_:


$$loss_{MF}=-\frac{\text{1}}{n}\sum_{i=1}^{n}\sum_{k=1}^{k}t_{ik}\log\left(y_{ik}\right)$$ (1)

For the cross entropy loss function mentioned above, *n* denotes the number of feature maps; the variable *y*_*ik*_ represents the probability that the i-th feature map belongs to the k-th category. If the i-th feature map belongs to the k-th category, the variable *t*_*ik*_ = 1; otherwise, *t*_*ik*_ = 0. As for OSNR estimation, it is a regression task, and its loss function is the mean square error loss function *loss*_*OSNR*_:


$$loss_{OSNR}=\frac{\text{1}}{n}{\sum_{i=1}^{n}\left(y_{i}-\overset{\wedge }{y_{i}}\right)}^{\text{2}}$$ (2)

$y_{i}$ and $\overset{\wedge }{y_{i}}$ are the true and predicted values of the OSNR corresponding to the i-th feature map, respectively. Assuming that the weights of the loss functions for MF and OSNR are $\lambda_{\text{1}}$ and $\lambda_{\text{2}}$, respectively, the total loss function of the model is denoted as *loss*_*all*_:


$$loss_{all}=\lambda_{\text{1}}loss_{MF}+\lambda_{\text{2}}loss_{OSNR}$$ (3)

### 3.2 Radon transform

**Fig 1:**
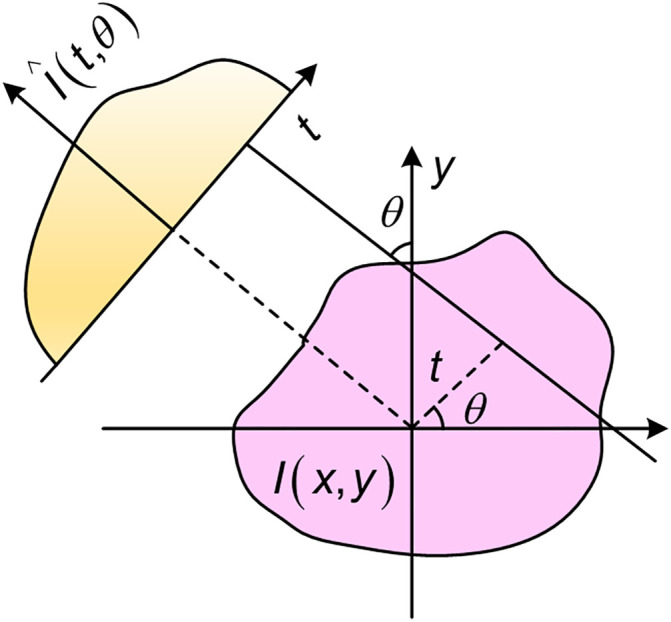
The principle of Radon transformation.

The Radon transform can be used to detect linear features in an image and analyze its geometric structure. Its core idea is to project the image in different directions, converting two-dimensional image information into one-dimensional projection data for image feature extraction. Let us assume that the two-dimensional function I(x,y) represents a constellation diagram image. When applying the Radon transform R(t,θ) to it, we have [28,29]:


$$R\left(t,\theta \right)=\int_{-\infty }^{\infty }\int_{-\infty }^{\infty }I\left(x,y\right)\delta \left[t-x\cos\;(\theta )-y\sin\;(\theta )\right]dxdy$$ (4)

where, δ(·) represents the Dirac function, *t* denotes the normal distance from *t*he origin to the projection line, and *θ* signifies the projection angle. This angle is defined as the angle between the normal distance and the horizontal axis of the image, as illustrated in Fig 1.

**Fig 2:**
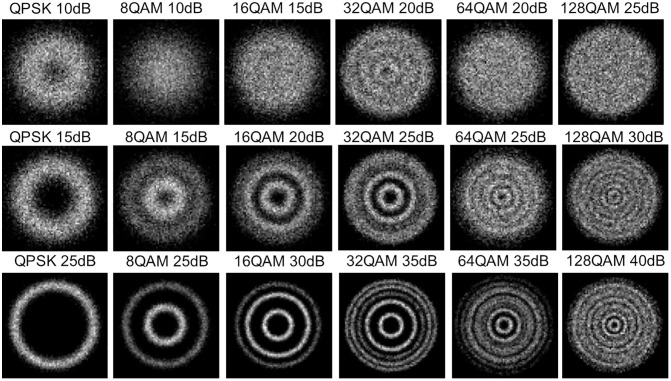
Ring constellation diagram.

A single-mode optical fiber is configured with a length of 160 km, corresponding to a dispersion of 2560 ps/nm and a differential group delay of 16 ps. The signal is transmitted through this optical fiber, undergoing dispersion compensation and equalization via the constant modulus algorithm. Coherent detection is then performed using a coherent receiver with a phase deviation of 5° and a frequency offset of 10 MHz. Scatter density constellation diagrams of six mainstream MFs are obtained, as presented in Fig 2. It can be observed that the constellation diagrams of different MFs exhibit distinct differences in the number of rings and their radius sizes. With the increase in OSNR, the brightness of the same ring for the same type of MF becomes clearer, which is conducive to realizing the tasks of MF identification and OSNR estimation.

**Fig 3:**
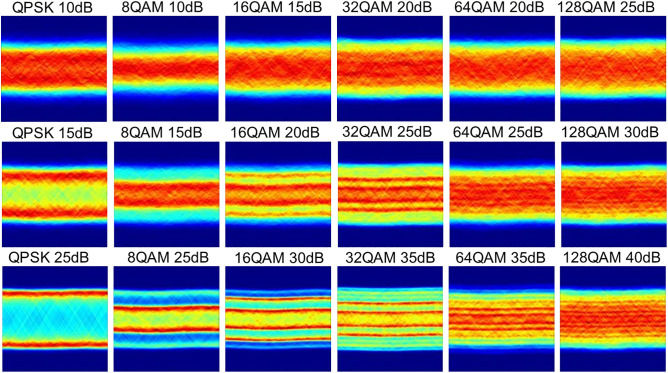
Radon transform of the constellation diagram.

Constellation diagrams were constructed based on the I/Q baseband data obtained from quadrature demodulation, where the in-phase component (I channel) served as the abscissa and the quadrature component (Q channel) as the ordinate. Scatter density plots were generated using the kernel density estimation method, as illustrated in Fig 2. Since the extracted constellation diagrams do not exhibit ideal symmetry, the projection angle was set to range from 0° to 360° when performing Radon transform on these constellation diagrams in this study, with the results presented in Fig 3. The Radon transform images reveal that at relatively high OSNR levels, the image features of different MFs show significant differences. However, at low OSNR levels, the distinctions in image features among 32QAM, 64QAM, and 128QAM become less pronounced, which may pose challenges for the MF recognition task.

### 3.3 Taylor score pruning

Taylor Pruning is a model compression technique that relies on Taylor Expansion to evaluate the importance of neural network parameters, thereby achieving model compression. Its core principle is to quantify the contribution of parameters to the model’s loss function and iteratively remove non-critical parameters (such as filters in convolutional layers) while maximizing the retention of model performance. Assume the loss function of the model is C(θ) (where θ represents the set of all parameters of the model), and let $h_{i}$ denote the filter parameter. The loss variation after removing $h_{i}$ can be expressed as [25]:


$$\Delta C\left(h_{i}\right)=C\left(D,h_{i}=0\right)-C\left(D,h_{i}\right)$$ (5)

Where $C\left(D,h_{i}=0\right)$ denotes the loss of the model on dataset *D* after removing $h_{i}$, and $C\left(D,h_{i}\right)$ represents the loss of the model on dataset *D* when retaining $h_{i}$. Directly calculating $C\left(D,h_{i}=0\right)$ requires re-performing forward propagation, which incurs high computational cost. Therefore, Taylor Pruning approximates $C\left(D,h_{i}=0\right)$ using first-order Taylor expansion, and it follows that:


$$C\left(D,h_{i}=0\right)\approx C\left(D,h_{i}\right)-\frac{\partial C}{\partial h_{i}}h_{i}$$ (6)

In the above equation, $\frac{\partial C}{\partial h_{i}}$ denotes the gradient of the loss function with respect to parameter $h_{i}$, which reflects the sensitivity of the loss function to changes in $h_{i}$.

Substitute equation (6) into equation (5) to obtain:


$$\left|\Delta C\left(h_{i}\right)\right|\approx \left|\frac{\partial C}{\partial h_{i}}h_{i}\right|$$ (7)

Equation (7) reflects the expected effect of the clipping filter parameter i on the loss function.

The physical interpretation of Equation (7) is that the expected impact of parameter $h_{i}$ on the loss function is approximately equal to the absolute value of the product of the gradient (of the loss function with respect to $h_{i}$ and parameter $h_{i}$ itself.

comprehensively evaluate the importance of an entire filter instead of individual parameters, Taylor Pruning calculates the average of the contributions from all parameters within the filter, yielding the Taylor criterion $\Theta_{TE}$:


$$\Theta_{TE}\left(z_{l,k}^{\left(m\right)}\right)=\frac{\text{1}}{M}\sum m=1M\left|\frac{\partial C}{\partial z_{l,k}^{\left(m\right)}}z_{l,k}^{\left(m\right)}\right|$$ (8)

Where $z_{l,m}^{\left(k\right)}$ denotes the *m*-th element of the flattened *k*-th feature map in the *l*-th layer. The feature map is generated by convo*l*ving the filter with the input, so the elements of the feature map exhibit a strong correlation with the filter parameters. *M* represents the total number of elements of the flattened feature map. By averaging the gradient-activation products of the feature map elements, this criterion quantifies the contribution of the entire filter to the loss—specifically, a smaller $\Theta_{TE}$ implies that the filter is less critical and thus more suitable for pruning.

**Fig 4:**
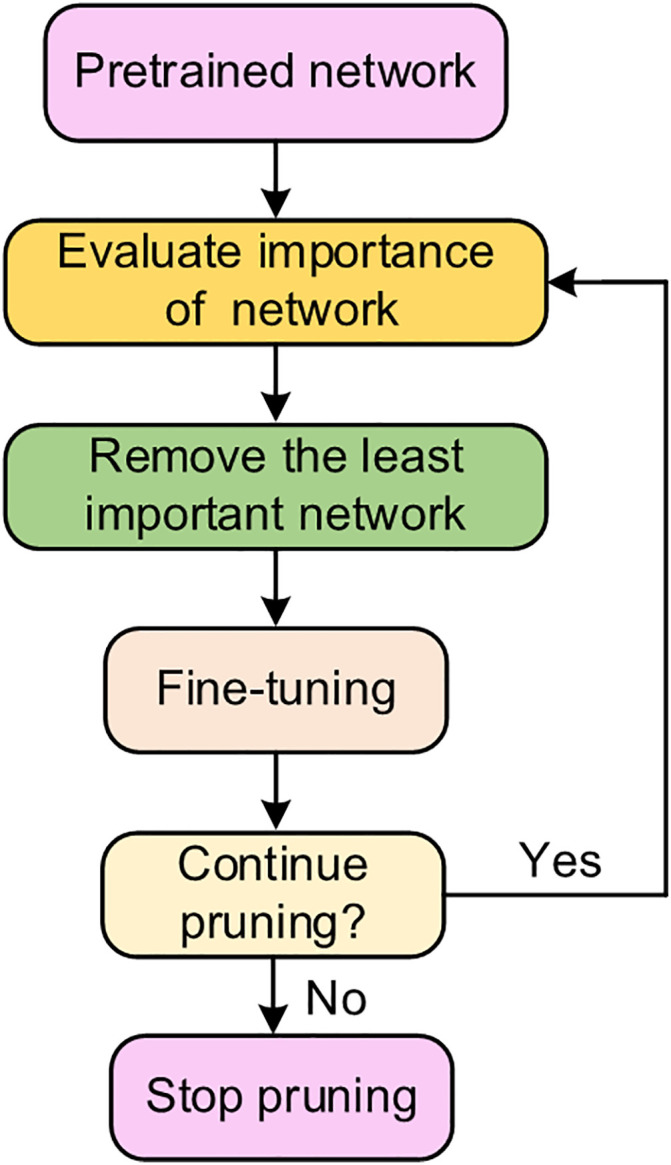
The process of Taylor score pruning.

The specific steps of the pruning process are illustrated in Fig 4 Taylor Pruning is an iterative approach that involves multiple pruning rounds with model fine-tuning performed after each round. The workflow is as follows:

(1) Pretraining and fine-tuning initialization: First, the neural network undergoes pretraining followed by fine-tuning on the target task until convergence, resulting in an “initial full-precision model” with all parameters retained.
(2) Taylor criterion calculation, sorting, and pruning: For the layers to be pruned in the model, such as convolutional layers, the Taylor criterion $\Theta_{TE}$ of each filter is calculated. These filters are then sorted in ascending order of $\Theta_{TE}$, where a smaller value indicates lower importance. Subsequently, an iterative pruning process is conducted to remove a certain proportion of the least critical filters.
(3) Fine-tuning for accuracy recovery: After each pruning round, the removal of model parameters causes a temporary decline in performance. Therefore, the pruned model requires re-fine-tuning, which involves training for a small number of epochs to re-optimize the remaining parameters and recover the accuracy lost due to pruning.
(4) Termination conditions: The pruning process stops when the model meets any of the following criteria: the model reaches the target compression ratio, such as a 40%–80% reduction in the number of parameters.
(5) Stopping Criterion: First, the compression ratio (*R*) is defined as shown in Equation (9), where *N*_2_ and *N*_1_ represent the total number of parameters after pruning and the total number of original parameters, respectively. The setting of the compression ratio in the stopping criterion is not merely intended to minimize the number of parameters; instead, it is derived by integrating considerations of training efficiency and model accuracy. When *R* is lower than a certain threshold, the reductions in parameters and FLOPs (floating-point operations) achieved via pruning, as well as the improvement in training speed, may be limited. When *R* exceeds a certain threshold, the model parameters are excessively pruned; even with multiple rounds of fine-tuning, the model accuracy may decrease significantly. Therefore, the compression ratio should be appropriately set by comprehensively considering training efficiency, model complexity, and model accuracy. In this paper, the compression ratio is set within the range of 40% to 80% to investigate the variations in model performance.

$$R=1-\frac{N_{\text{2}}}{N_{\text{1}}}$$ (9)

### 3.4 Self attention mechanism

The multi-head self-attention mechanism originates from the field of natural language processing. Its core idea is that when processing sequential data, attention weights are calculated and assigned to each position in the input sequence—this enables the model to more effectively capture critical information. Notably, this mechanism is equally applicable in the field of image processing.


$$Q=XW^{Q},K=XW^{K},V=XW^{V}$$ (10)

Among them, $W^{Q}$、$W^{K}$ and $W^{V}$ are learnable weight matrices used to map the input sequence X to the query, key, and value space. Therefore, single head-self-attention is defined as:


$$Attention\left(Q,K,V\right)=softmax\left(\frac{QK^{T}}{\sqrt{d_{k}}}\right)V$$ (11)

Among them, *Q* denotes the query matrix, *K*^*T*^ represents the transpose of the key matrix *K*, and $\sqrt{d_{k}}$ stands for the dimension of the key vector. The outputs of multiple single-head attention modules are concatenated, followed by a linear projection, to yield the final output of multi-head self-attention. The corresponding mathematical expression is given as follows:


$$MultiHead\left(Q,K,V\right)=Concat\left({head}_{\text{1}},{head}_{\text{2}},...,{head}_{h}\right)W^{O}$$ (12)

Among them, head_*i*_ = Attention(*Q*_*i*_, *K*_*i*_ and *V*_*i*_), *Q*_*i*_, *K*_*i*_ and *V*_*i*_ denote the i-th attention head, and the corresponding query, key, and value matrices for this head, respectively. *W*^*o*^ represents the linear transformation matrix for the final output projection. In this work, the number of attention heads is set to 2 to reduce the model complexity while ensuring sufficient feature capture capability.

## 4. System setup and network structure

### 4.1 Simulation setup

**Fig 5:**
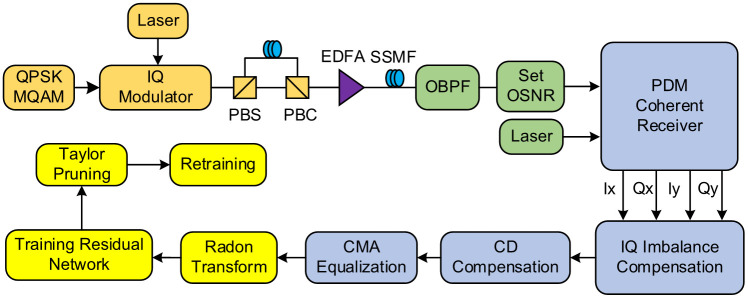
Schematic diagram of MF identification and OSNR estimation. EDFA: erbium-doped fiber amplifier; PBS: polarization beam splitter; PBC: polarization beam combiner; OBPF: optical band-pass filter; CD: chromatic dispersion; PDM: polarization division multiplexing; CMA: constant modulus algorithm.

To verify the joint monitoring performance of the proposed model for MF and OSNR, we constructed an optical communication simulation link using VPI Transmission Maker 11.1, as presented in Fig 5. At the transmitter, pseudo-random bit sequences were first mapped to generate baseband I/Q electrical signals corresponding to six modulation formats: QPSK, 8QAM, 16QAM, 32QAM, 64QAM, and 128QAM. A laser then output a continuous optical carrier, and an I/Q modulator loaded electrical domain information onto the phase and amplitude of the optical carrier via the electro-optic effect to generate modulated optical signals. These signals were amplified by an erbium-doped fiber amplifier before being transmitted into a standard single-mode fiber link. At the receiver, a coherent receiver performed coherent detection to obtain four I/Q signals (i.e., Ix, Qx, Iy, and Qy). Subsequently, dispersion compensation and constant modulus algorithm equalization (modulation-format independent) were conducted. Finally, the equalized I/Q signals were collected to generate scatter density constellation diagrams, and geometric projection features of the constellations were extracted via Radon transform as inputs to the neural network. Additionally, pruning and fine-tuning were sequentially performed on the neural network to reduce its complexity.

### 4.2 Model structure

**Fig 6:**
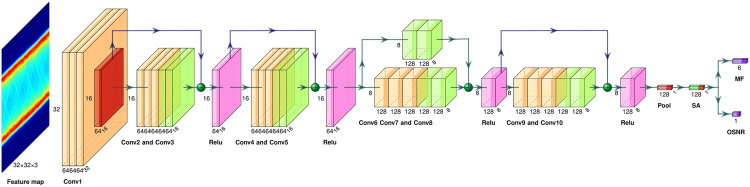
SA-ResNet model structure. Conv, convolution; FC, full connection; SA, self-attention mechanism.

ResNet18, a classic residual network, effectively addresses the challenges of vanishing and exploding gradients in deep network training by incorporating residual connections, thereby ensuring stable training of deep networks. In comparison to more complex models like ResNet50/101, ResNet18 features a simpler structure and lower computational demands [30]. To further streamline the model, we have simplified the design of ResNet18, resulting in a modified version termed SA-ResNet, as illustrated in Fig 6 SA-ResNet serves as a multi-task learning model, with MF recognition as a classification task and OSNR estimation as a regression task. Key modifications include resizing the input feature map from 224 × 224 × 3–32 × 32 × 3 and removing redundant residual modules. The revised structure comprises 10 filter groups (denoted as Conv1 to Conv10): Conv1, Conv2, Conv4, Conv6, and Conv9 each consist of convolutional layers, batch normalization layers, and ReLU layers; Conv3, Conv5, Conv7, Conv8, and Conv10 contain only convolutional layers and batch normalization layers. Following the global pooling layer, we integrated an attention mechanism with an output channel size of 128, which features two heads where the key, query, and value channels are all set to 128. Consequently, the total number of model parameters was significantly reduced from 11.6 M (original ResNet18) to 750.9 K in the modified SA-ResNet.

## 5. Results and discussion

In this chapter, Section 5.1 investigates and discusses the impact of different sample lengths and image resolutions on the (MF and OSNR monitoring performance of SA-ResNet. Section 5.2 explores the application of Taylor pruning to SA-ResNet, analyzes the influence of different compression ratios on model performance, and conducts a complexity analysis. Section 5.3 analyzes the robustness of SA-ResNet. Notably, in Sections 5.1 and 5.2, the dataset is randomly shuffled, with the training, validation, and test sets divided in a ratio of 70%, 15%, and 15%, respectively. In contrast, Section 5.3 adopts 5-fold cross-validation to evaluate the generalization ability of the model.

### 5.1 Monitoring performance of SA-ResNet

We used the optical fiber communication link shown in Fig 5 to generate the dataset. The settings of key parameters (e.g., laser source, fiber length, OSNR range for different MFs) are shown in Table 1. It is worth noting that since the actual devices are not ideal but have a certain phase deviation, both 90° optical mixers are set with a 5° phase offset. The fiber length was set to 160 km, with the chromatic dispersion and differential group delay within the fiber configured as 2560 ps/nm and 16 ps, respectively. While keeping other physical parameters unchanged, 100 different pseudo-random bit sequences are generated at the transmitting end through random number seeds, enabling each modulation format to transmit different symbolic information under the same OSNR condition. Therefore, our dataset contained a total of 9,600 images, calculated as follows: 6 MFs, 16 OSNR values per MF, and 100 samples per (MF, OSNR) pair (6 × 16 × 100 = 9,600).

**Table 1:** Simulation Parameter Settings.

Main parameters	Description
laser line width	100kHz
laser emission power	0dBm
frequency offset	10MHz
phase deviation	5°
Symbol rate	28 GBuad
optical fiber length	160km
dispersion coefficient	16 ps/nm/km
differential group delay	0.1ps/km
modulation format	QPSK/8QAM/16QAM/32QAM/64QAM/128QAM
OSNR range (QPSK)	10-25dB, step = 1dB
OSNR range (8QAM)	10-25dB, step = 1dB
OSNR range (16QAM)	15-30dB, step = 1dB
OSNR range (32QAM)	20-35dB, step = 1dB
OSNR range (64QAM)	20-35dB, step = 1dB
OSNR range (128QAM)	25-40dB, step = 1dB

**Fig 7:**
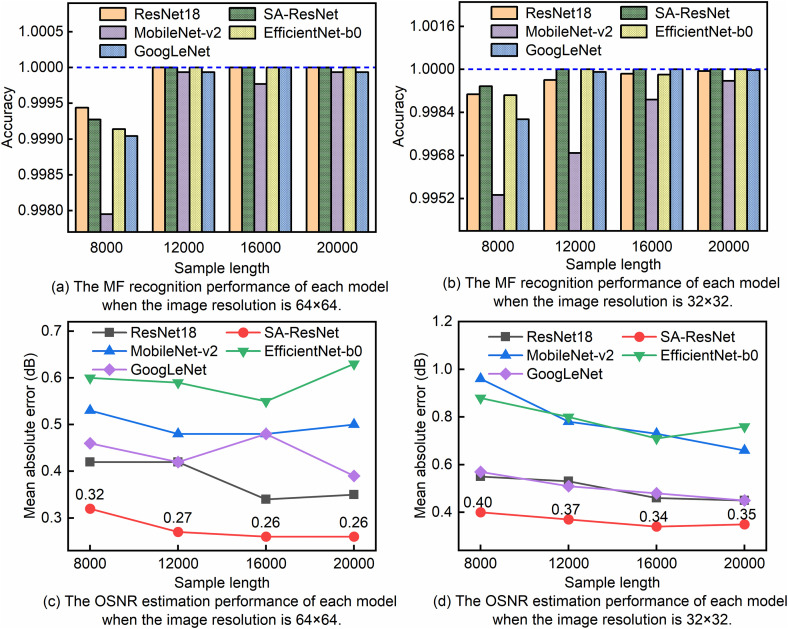
The monitoring performance of different models under different image resolutions and sample lengths.

The scientific and reasonable division of the original dataset is a key prerequisite for ensuring the effectiveness of model training and the reliability of model evaluation [3,18,31]. The dataset was randomly shuffled and split into a training set, test set, and validation set at a ratio of 70%, 15%, and 15%. L2 regularization was used to suppress overfitting. The weights of the MF classification task and the OSNR estimation regression task were set to 1:1. To study the monitoring performance of SA-ResNet under different sample lengths and image resolutions and to facilitate comparison, SA-ResNet, ResNet18, GoogLeNet, MobileNet-v2, and EfficientNet-b0 were trained for 200 epochs. Their performance on the test set is shown in Fig 7. When the image resolution was set to 64 × 64, SA-ResNet, ResNet18, and EfficientNet-b0 achieved the best MF recognition performance. Their accuracy reached 100% when the sample length was between 12,000 and 20,000. When the image resolution was set to 32 × 32, only SA-ResNet achieved 100% MF recognition accuracy with a sample length between 12,000 and 20,000, as shown in Figs 7(a) to 7(b). Within the sample length range of 8,000–20,000, SA-ResNet had a lower average OSNR estimation error than other models, regardless of whether the image resolution was 64 × 64 or 32 × 32. This is shown in Figs 7(c) to 7(d). Overall, for SA-ResNet, higher image resolution and longer sample length led to lower overall OSNR estimation error and higher MF recognition accuracy. To simplify the analysis, in the following study, we set the sample lengths to 16,000 and 20,000.

**Fig 8:**
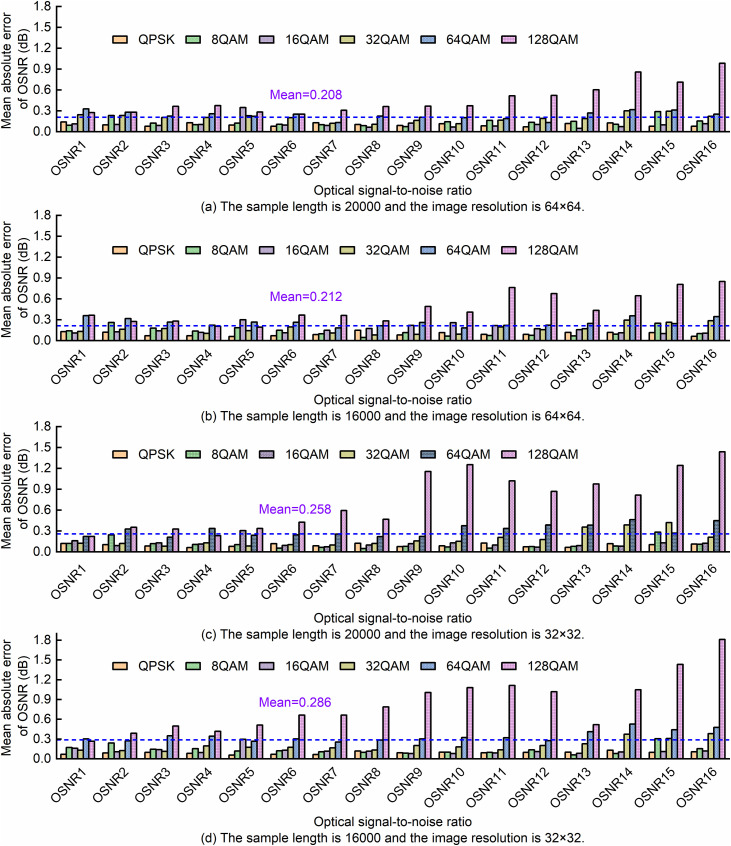
The SA-ResNet prediction errors of different MFs under different OSNR conditions.

The six types of MFs have 16 different OSNR values, with the range shown in Table 1; all are denoted as OSNR1 to OSNR16. Fig 8 presents the average absolute error of SA-ResNet for each OSNR estimation. The dotted lines in Fig 8 represent the mean values of the mean absolute errors for four scenarios (64 × 64/20,000 samples, 64 × 64/16,000 samples, 32 × 32/20,000 samples, 32 × 32/16,000 samples), which are 0.208 dB, 0.212 dB, 0.258 dB, and 0.286 dB, respectively. Notably, 128QAM exhibits the largest overall OSNR estimation error—especially at 32 × 32 resolution and OSNR > OSNR9 (33 dB)—due to relatively small differences in its constellation diagrams across OSNR levels (as visually confirmed in Fig 2).

**Fig 9:**
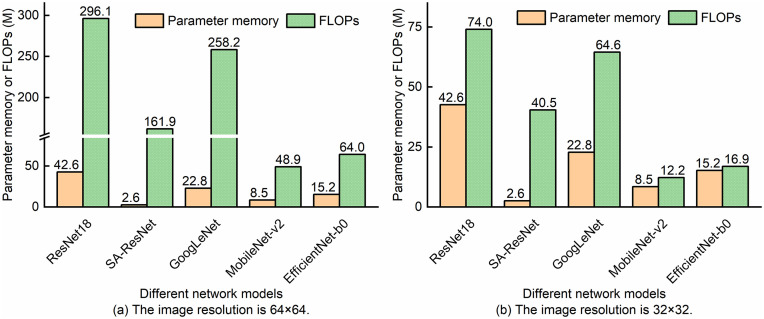
Complexity analysis of each model.

While SA-ResNet demonstrated promising comprehensive monitoring performance, analyzing its computational complexity is crucial for evaluating its feasibility in practical deployments. Accordingly, we analyzed the parameter memory and FLOPs of SA-ResNet, with the results presented in Fig 9. Among the compared models, SA-ResNet had the smallest parameter memory (2.6 M). However, its FLOPs were relatively high (161.9 M) at an image resolution of 64 × 64, which decreased significantly to 40.5 M at 32 × 32. Although SA-ResNet had higher FLOPs than MobileNet-v2 and EfficientNet-b0 at 32 × 32, it achieved the best performance in both MF recognition and OSNR estimation. Thus, SA-ResNet retained a distinct advantage. Subsequent discussions focus on the 32 × 32 resolution scenario; we further reduced FLOPs by compressing SA-ResNet via Taylor pruning to lower its computational complexity.

### 5.2 Monitoring performance of Taylor scores pruning

**Fig 10:**
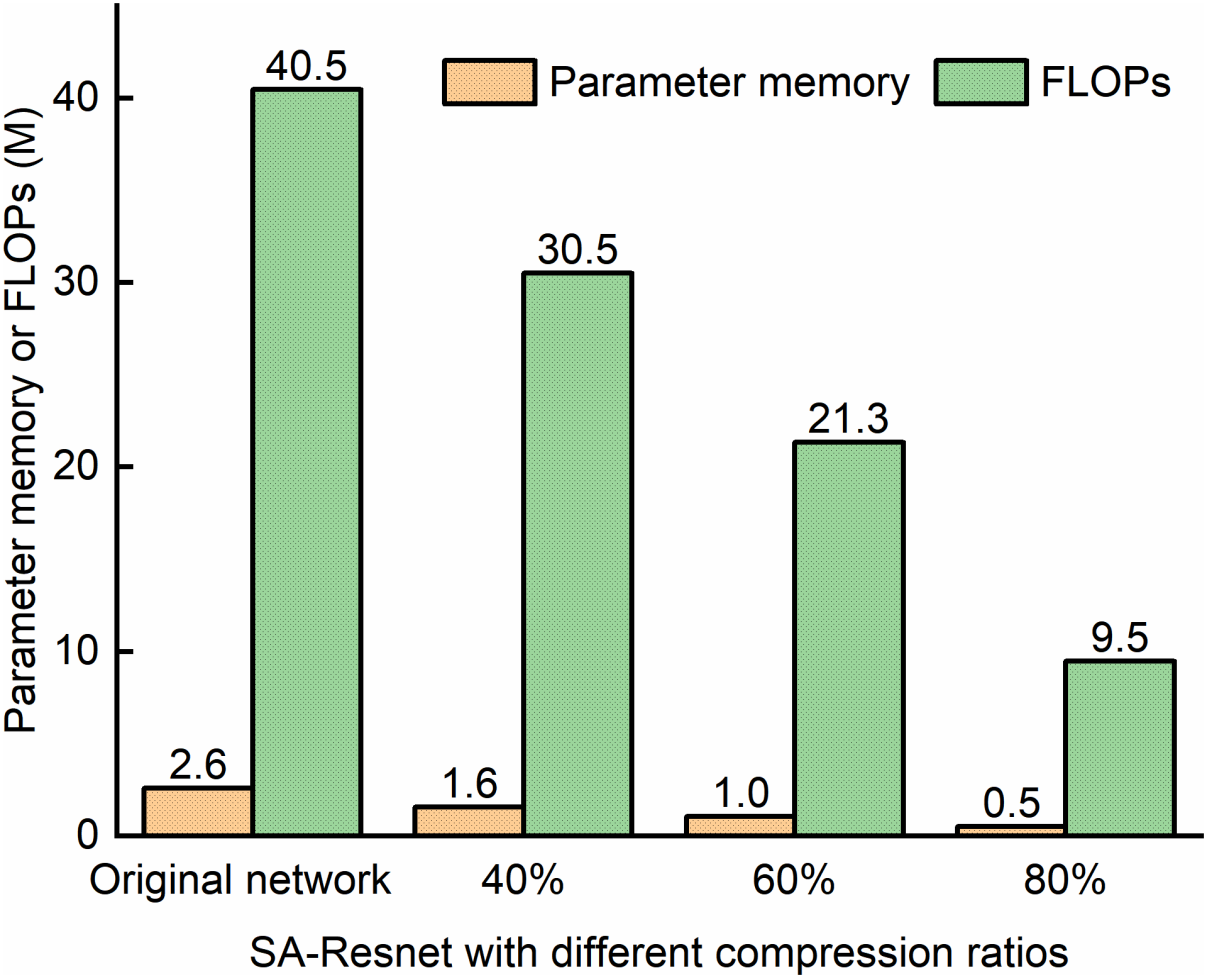
The model complexity of SA-ResNet under different compression ratios.

Taylor pruning is applied to SA-ResNet to identify and remove redundant parameters in order to reduce the model complexity. The SA-ResNet model has a total of 775 prunable filters. In the network pruning experiment, the maximum number of filters to be pruned in each iteration was set to 8. Compression ratios of 40%, 60%, and 80% were used as the termination conditions for SA-ResNet pruning. The original SA-ResNet had a parameter memory of 2.6 M and FLOPs of 40.5 M; after pruning, these metrics were reduced to 1.6 M, 1.0 M, 0.5 M (parameter memory) and 30.5 M, 21.3 M, 9.5 M (FLOPs), corresponding to parameter memory reductions of 38%, 62%, and 81%, and FLOPs reductions of 25%, 47%, and 77%, respectively. The corresponding FLOPs were 30.5M, 21.3M and 9.5M respectively, and the FLOPs were decreased by 25%, 47% and 77% respectively. This indicates that Taylor pruning can effectively reduce parameter memory and FLOPs, as shown in Fig 10.

**Fig 11:**
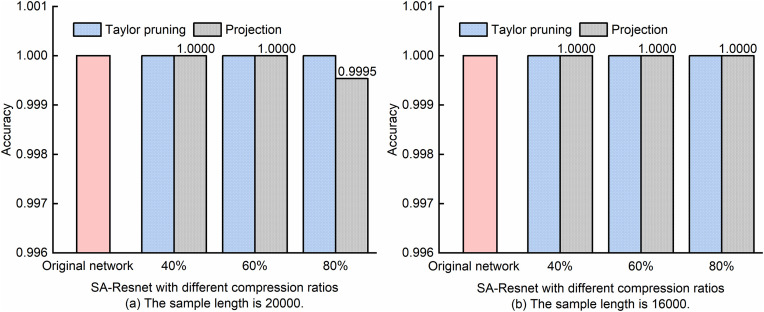
Comparison of SA-ResNet MF monitoring performance before and after model compression with fine-tuning.

**Fig 12:**
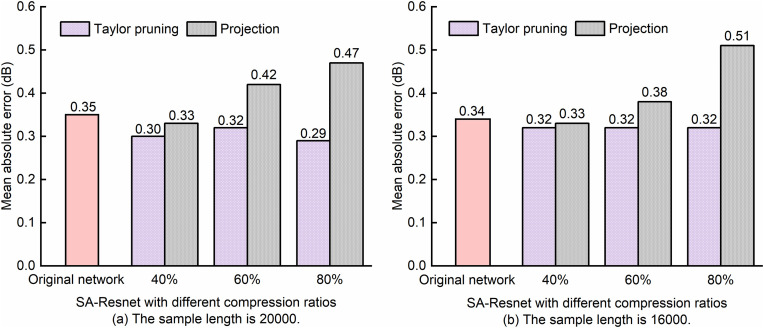
Comparison of SA-ResNet OSNR monitoring performance before and after model compression with fine-tuning.

It is worth noting that after Taylor pruning, the prediction performance of the model will decline, and fine-tuning is still needed to restore the model’s prediction accuracy. A similar model compression technique is projection, which uses principal component analysis of neuron activation values to retain key subspaces. It can not only precisely control the compression ratio according to the target memory, but also restore the network accuracy to nearly the original level after fine-tuning, balancing compression efficiency and model performance [32]. To verify the effectiveness of the adopted scheme, we used projection for monitoring performance comparison, and all the compressed models underwent 50 rounds of fine-tuning. The results are shown in Fig 11 and Fig 12. In this article, the “Original Network” all represents the unpruned SA-ResNet. The SA-ResNet using the Taylor pruning method, after fine-tuning, maintains a MF recognition accuracy of 100% regardless of whether the sample length is 16,000 or 20,000. However, when the projection algorithm is used with a sample length of 20,000 and a compression ratio of 80%, the MF recognition accuracy was 99.95%, as shown in Fig 11.

It can be seen from Fig 12 that after pruning SA-ResNet via Taylor pruning and fine-tuning, the mean absolute error range of OSNR is 0.29 dB to 0.30 dB when the sample length is 20,000, and it is consistently 0.32 dB when the sample length is 16,000. From this, it can be concluded that with a compression ratio of 40% to 80%, the mean absolute error of OSNR estimated by the fine-tuned model is lower than that of the original SA-ResNet, regardless of whether the sample length is 16,000 or 20,000. However, when the compression ratio ranges from 60% to 80%, although the projection-compressed model has been fine-tuned, the mean absolute error of OSNR estimated by the model is higher than that of the original SA-ResNet. When the compression ratio reaches 80%, the projection-compressed model exhibits significantly higher mean absolute errors for OSNR estimation (0.47 dB for 20,000 samples and 0.51 dB for 16,000 samples) compared to the original SA-ResNet. This is attributed to the fact that projection compression retains key subspaces via principal component analysis; however, when compression ratios exceed 60%, it may discard critical fine-grained features of constellation diagrams—especially for high-order modulation formats (e.g., 128QAM)—which in turn leads to degraded estimation accuracy.

### 5.3 Robustness analysis

**Fig 13:**
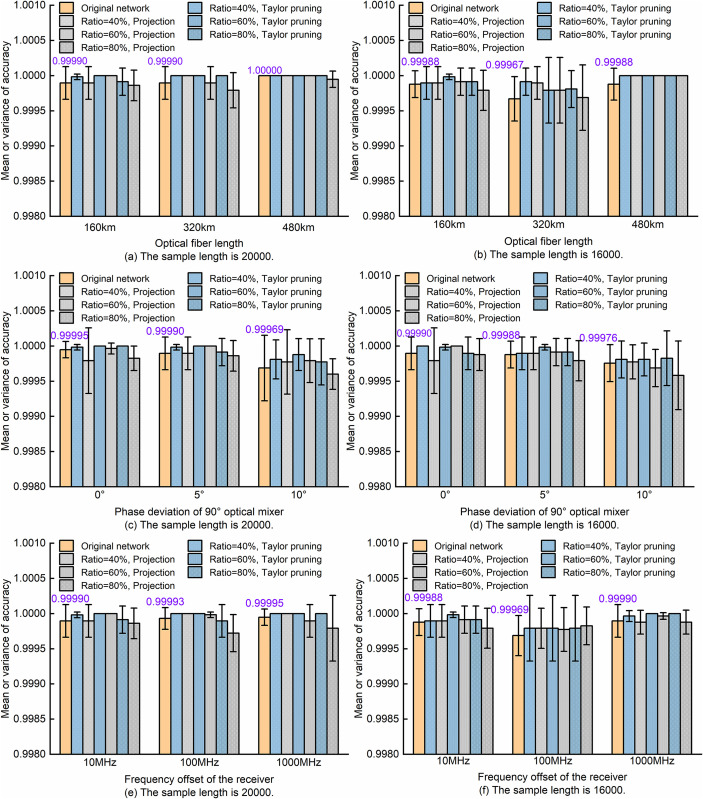
MF monitoring performance with different link parameters.

**Fig 14:**
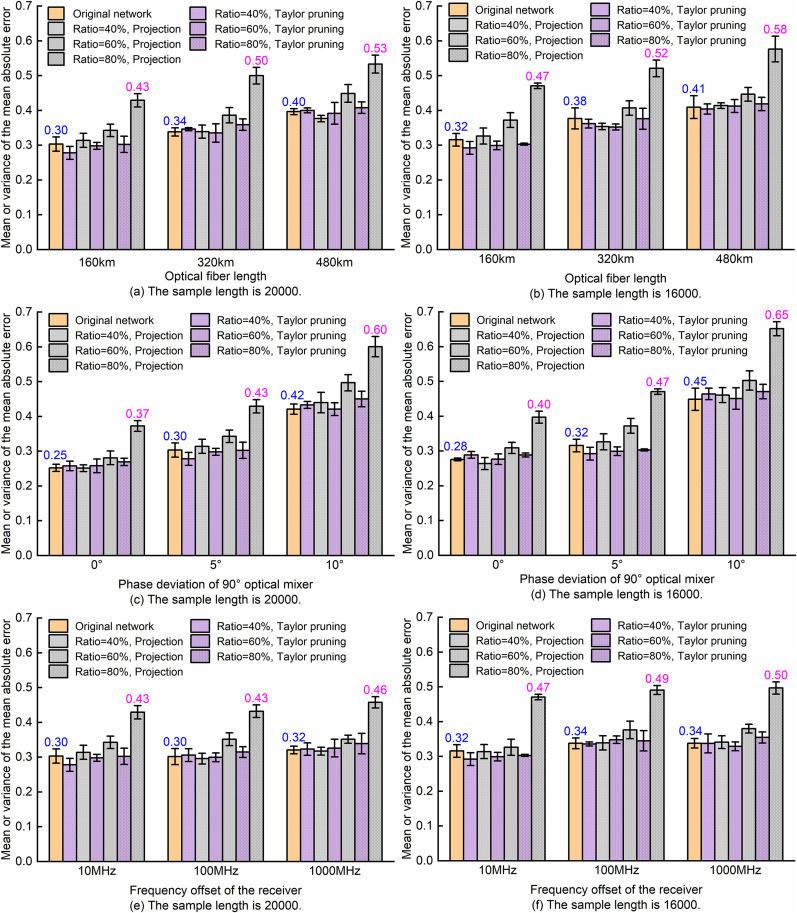
OSNR monitoring performance with different link parameters.

The dataset for the above experiment is derived from the optical fiber link in Fig 5. The length of the optical fiber, the phase offset of the 90° optical mixer, and the frequency offset at the receiver are all fixed. Therefore, it is necessary to study the impact of changes in these key parameters on SA-ResNet. In addition, to avoid deviations in the model evaluation results due to differences in the sequence of training data, we adopted 5-fold cross-validation to evaluate the model’s robustness [33–36]. We used 5-fold cross-validation to evaluate the model, and the analysis results obtained are shown in Fig 13 to Fig 14. The bar charts in the figures represent the mean, and the error bars represent the standard deviation. All subgraphs in Figs 13 to 14 share the same vertical coordinate scale. If the error bar is not shown in the graph, it means that the value of the standard deviation is 0.

Figs 13 and 14 show the comparison of the 5-fold cross-validation results of MF recognition and OSNR estimation under different link parameters. From the bar chart in Fig 13, it is observed that across various sample lengths (16000 and 20000), fiber lengths (160 km, 320 km, 480 km), mixer phase offsets (0°, 5°, 10°) and frequency offsets (10 MHz, 100 MHz, 1000 MHz), the average MF recognition accuracy of all networks exceeds 99.90%. Comparing the subgraphs in Fig 13, it can be seen that the overall MF recognition performance of the SA-ResNet after Taylor pruning and that after projection compression shows little difference. However, under the condition of a higher compression ratio, the decline trend of the MF recognition performance of the SA-ResNet model after Taylor pruning is slightly smaller than that of the SA-ResNet with projection compression. That is, when the compression ratio is 80%, the MF recognition performance of the Taylor pruned model is generally better than that of the projection-compressed model.

A key point to note is that the original networks presented in Fig 13(a)~(b) and Fig 14(a)~(b) rely on datasets collected under defined link parameters: 160 km fiber length, an optical 90° mixer with 5° phase deviation, and a receiver with 10 MHz frequency deviation. These datasets are the same as those used in Fig 7 above. For sample lengths of 20,000 and 16,000, the cross-validated MF recognition accuracy decreased from the single-run fine-tuning accuracy of 100.000% to 99.990% ± 0.023% and 99.988% ± 0.019%, respectively. Corresponding OSNR estimation mean absolute errors decreased from the original 0.35 dB and 0.34 dB to 0.30 ± 0.02 dB and 0.32 ± 0.02 dB. These results suggest that SA-ResNet exhibits decent monitoring performance. Here, the mean ± standard deviation is used to represent the cross-validation results. It should be particularly noted that the highest recognition accuracy rate of MF is 100%, which is the same in the following text and will not be elaborated further.

As can be seen from the bar chart in Fig 14, with the increase of fiber length, the angle of phase deviation of the mixer and the frequency offset of the receiver, the average error of OSNR estimation in the original network and compressed model generally shows an upward trend. Under the variations of these physical parameters, the OSNR estimation error ranges of the Taylor pruning and projection compression models are respectively: 0.26 ± 0.01dB to 0.47 ± 0.02dB and 0.25 ± 0.01dB to 0.65 ± 0.02dB; When the physical parameters of fiber length, phase deviation and frequency offset are the same, with the increase of compression ratio, the upward trend of OSNR estimated by the Taylor-pruned SA-ResNet is not obvious relative to that estimated by the original SA-ResNet. In contrast, the model of projection compression will increase significantly, especially when the compression ratio is 80%.

In conclusion, as the physical parameters of optical fiber length, phase deviation and frequency offset increase, the accuracy of MF recognition does not show a simple positive or negative correlation. However, the OSNR estimation error exhibits a general positive correlation with increasing fiber length, mixer phase offset, and receiver frequency offset. In addition, Taylor pruning can be overall superior to the projection algorithm in terms of MF and OSNR monitoring performance at a high compression rate of 80%.

Due to the limitations of experimental conditions, in the above analysis and discussion, the datasets used were all generated using the VPI Transmission Maker simulation software. It should be noted that there may be a certain deviation between the simulation data and the measured data in real scenarios, and this deviation may have an impact on the performance evaluation results of the model. Furthermore, in terms of the evaluation dimension of model complexity, this study temporarily focuses on two core indicators: parameter storage overhead and FLOPs. However, in actual engineering deployment scenarios, the implementation effect of the model still needs to comprehensively consider key factors such as the underlying optimization features of deep learning frameworks and the architectural adaptability of hardware platforms. In addition, the approach adopted in our work for feature map processing is relatively simplistic. The integration of singular spectrum analysis may yield positive effects [37–40].

## 6. Conclusion

This study proposes a residual network integrated with a self-attention mechanism (termed SA-ResNet) to process Radon-transformed constellation diagrams for joint MF recognition and OSNR estimation. Key findings include: (1) At 32 × 32 resolution, 16,000 samples, and 160 km fiber length, SA-ResNet achieved 100% MF recognition accuracy and an OSNR estimation mean absolute error of 0.34 dB, with 2.6 M parameters and 40.5 M FLOPs; 5-fold cross-validation confirmed robust performance, with 99.988% ± 0.019% MF accuracy and 0.32 ± 0.02 dB OSNR mean absolute error; (2) Taylor pruning reduced parameters to 0.5–1.6 M and FLOPs to 9.5–30.5 M (40%–80% compression), while maintaining >99.90% average MF accuracy and 0.26–0.47 dB OSNR mean absolute error across varying link parameters (160–480 km fiber, 0°–10° phase offset, 10–1000 MHz frequency offset). These results demonstrate that Taylor-pruned SA-ResNet balances low computational complexity and high monitoring performance, thus holding certain potential for lightweight deployment in resource-constrained optical network edge nodes.

## Supporting information

S1 Dataset. (DOCX)
