# Supplementary material for: A modulation format recognition and optical signal-to-noise ratio monitoring scheme based on residual network and Taylor score pruning
Source: PLoS One. 2025 Oct 13;20(10):e0333936. doi: 10.1371/journal.pone.0333936 (PMC12517532; doi:10.1371/journal.pone.0333936)
Supplement: S1 — (DOCX) [file pone.0333936.s001.docx]

**Figure 7 Original data set**

| Accuracy, image resolution: 64 × 64 | | | | | |
| --- | --- | --- | --- | --- | --- |
| Sample length | ResNet18 | SA-ResNet | MobileNet-v2 | EfficientNet-b0 | GoogLeNet |
| 8000 | 0.99944 | 0.99927 | 0.99795 | 0.99914 | 0.99904 |
| 12000 | 1 | 1 | 0.99993 | 1 | 0.99993 |
| 16000 | 1 | 1 | 0.99977 | 1 | 1 |
| 20000 | 1 | 1 | 0.99993 | 1 | 0.99993 |
| Accuracy, image resolution: 32 × 32 | | | | | |
| 8000 | 0.99907 | 0.99937 | 0.99534 | 0.99904 | 0.99815 |
| 12000 | 0.9996 | 1 | 0.99689 | 1 | 0.9999 |
| 16000 | 0.99984 | 1 | 0.99888 | 0.9998 | 1 |
| 20000 | 0.99993 | 1 | 0.99957 | 1 | 0.99997 |
| Mean absolute error, image resolution: 64 × 64 | | | | | |
| 8000 | 0.42 | 0.32 | 0.53 | 0.6 | 0.46 |
| 12000 | 0.42 | 0.27 | 0.48 | 0.59 | 0.42 |
| 16000 | 0.34 | 0.26 | 0.48 | 0.55 | 0.48 |
| 20000 | 0.35 | 0.26 | 0.5 | 0.63 | 0.39 |
| Mean absolute error, image resolution: 32 × 32 | | | | | |
| 8000 | 0.55 | 0.4 | 0.96 | 0.88 | 0.57 |
| 12000 | 0.53 | 0.37 | 0.78 | 0.8 | 0.51 |
| 16000 | 0.46 | 0.34 | 0.73 | 0.71 | 0.48 |
| 20000 | 0.45 | 0.35 | 0.66 | 0.76 | 0.45 |

**Figure 8 Original data set**

| Mean absolute error, sample length=20000, image resolution=64 × 64 | | | | | | |
| --- | --- | --- | --- | --- | --- | --- |
| OSNR | QPSK | 8QAM | 16QAM | 32QAM | 64QAM | 128QAM |
| OSNR1 | 0.1405 | 0.09389 | 0.11259 | 0.24525 | 0.32753 | 0.27431 |
| OSNR2 | 0.0977 | 0.23362 | 0.10506 | 0.2337 | 0.28096 | 0.28066 |
| OSNR3 | 0.07729 | 0.12331 | 0.08937 | 0.20536 | 0.22473 | 0.36516 |
| OSNR4 | 0.12769 | 0.09957 | 0.10259 | 0.2054 | 0.25897 | 0.37588 |
| OSNR5 | 0.09424 | 0.12252 | 0.34899 | 0.23109 | 0.22289 | 0.28386 |
| OSNR6 | 0.0787 | 0.10655 | 0.09509 | 0.20271 | 0.25123 | 0.25261 |
| OSNR7 | 0.13014 | 0.10095 | 0.08932 | 0.12188 | 0.13159 | 0.30877 |
| OSNR8 | 0.10245 | 0.08797 | 0.06526 | 0.10759 | 0.22499 | 0.36246 |
| OSNR9 | 0.08924 | 0.07335 | 0.12452 | 0.16138 | 0.20758 | 0.36689 |
| OSNR10 | 0.11503 | 0.14275 | 0.06598 | 0.11402 | 0.20085 | 0.37245 |
| OSNR11 | 0.08418 | 0.16198 | 0.08158 | 0.16419 | 0.18729 | 0.51728 |
| OSNR12 | 0.06947 | 0.1349 | 0.10432 | 0.19138 | 0.13156 | 0.52025 |
| OSNR13 | 0.11728 | 0.14781 | 0.04893 | 0.18715 | 0.26898 | 0.60262 |
| OSNR14 | 0.12673 | 0.10749 | 0.07258 | 0.30069 | 0.31589 | 0.85656 |
| OSNR15 | 0.07814 | 0.28817 | 0.09876 | 0.29555 | 0.31247 | 0.71195 |
| OSNR16 | 0.07852 | 0.15327 | 0.11463 | 0.22117 | 0.25354 | 0.98424 |
| Mean absolute error, sample length=16000, image resolution=64 × 64 | | | | | | |
| OSNR | QPSK | 8QAM | 16QAM | 32QAM | 64QAM | 128QAM |
| OSNR1 | 0.1301 | 0.14131 | 0.11068 | 0.13236 | 0.35839 | 0.36561 |
| OSNR2 | 0.12099 | 0.25966 | 0.12581 | 0.16143 | 0.31816 | 0.27432 |
| OSNR3 | 0.06912 | 0.17962 | 0.14081 | 0.17295 | 0.26576 | 0.28071 |
| OSNR4 | 0.06927 | 0.13755 | 0.11669 | 0.10439 | 0.22111 | 0.2033 |
| OSNR5 | 0.05949 | 0.18409 | 0.29998 | 0.14407 | 0.26646 | 0.19469 |
| OSNR6 | 0.06936 | 0.14726 | 0.11226 | 0.19632 | 0.2632 | 0.36626 |
| OSNR7 | 0.08626 | 0.09936 | 0.14913 | 0.1101 | 0.18101 | 0.36257 |
| OSNR8 | 0.14817 | 0.04423 | 0.17409 | 0.08115 | 0.21286 | 0.28191 |
| OSNR9 | 0.08219 | 0.11411 | 0.21755 | 0.08921 | 0.26125 | 0.4903 |
| OSNR10 | 0.11588 | 0.06376 | 0.25853 | 0.09387 | 0.18166 | 0.40897 |
| OSNR11 | 0.0895 | 0.07329 | 0.21686 | 0.19754 | 0.21832 | 0.76366 |
| OSNR12 | 0.08962 | 0.07687 | 0.17207 | 0.15802 | 0.22245 | 0.67599 |
| OSNR13 | 0.11677 | 0.06657 | 0.1564 | 0.16997 | 0.2468 | 0.43409 |
| OSNR14 | 0.11809 | 0.09211 | 0.11082 | 0.29323 | 0.35743 | 0.64501 |
| OSNR15 | 0.11541 | 0.25046 | 0.10216 | 0.26461 | 0.243 | 0.80886 |
| OSNR16 | 0.06115 | 0.09955 | 0.10632 | 0.28548 | 0.34459 | 0.84913 |
| Mean absolute error, sample length=20000, image resolution=32× 32 | | | | | | |
| OSNR | QPSK | 8QAM | 16QAM | 32QAM | 64QAM | 128QAM |
| OSNR1 | 0.12084 | 0.12169 | 0.15946 | 0.12222 | 0.22055 | 0.22108 |
| OSNR2 | 0.10275 | 0.24465 | 0.09004 | 0.12105 | 0.329 | 0.35252 |
| OSNR3 | 0.08347 | 0.11791 | 0.12995 | 0.08081 | 0.21106 | 0.32725 |
| OSNR4 | 0.06047 | 0.10547 | 0.11235 | 0.12821 | 0.33514 | 0.23151 |
| OSNR5 | 0.07901 | 0.10501 | 0.30459 | 0.08544 | 0.23898 | 0.3352 |
| OSNR6 | 0.11637 | 0.05281 | 0.09229 | 0.1045 | 0.24331 | 0.4268 |
| OSNR7 | 0.08658 | 0.06507 | 0.07197 | 0.09953 | 0.25494 | 0.59452 |
| OSNR8 | 0.12395 | 0.04884 | 0.09696 | 0.12156 | 0.21808 | 0.46765 |
| OSNR9 | 0.0771 | 0.07863 | 0.1167 | 0.15575 | 0.22124 | 1.15432 |
| OSNR10 | 0.08749 | 0.07148 | 0.13036 | 0.15003 | 0.37648 | 1.2542 |
| OSNR11 | 0.12727 | 0.05272 | 0.09901 | 0.20727 | 0.33715 | 1.02173 |
| OSNR12 | 0.07401 | 0.07443 | 0.06855 | 0.17741 | 0.38677 | 0.8681 |
| OSNR13 | 0.06491 | 0.07926 | 0.09066 | 0.35734 | 0.38346 | 0.97603 |
| OSNR14 | 0.11711 | 0.08419 | 0.08052 | 0.38679 | 0.46355 | 0.81682 |
| OSNR15 | 0.10399 | 0.28419 | 0.128 | 0.42002 | 0.26817 | 1.24236 |
| OSNR16 | 0.11348 | 0.1084 | 0.12374 | 0.21162 | 0.44866 | 1.43783 |
| Mean absolute error, sample length=16000, image resolution=32 × 32 | | | | | | |
| OSNR | QPSK | 8QAM | 16QAM | 32QAM | 64QAM | 128QAM |
| OSNR1 | 0.06689 | 0.17102 | 0.16072 | 0.1315 | 0.30072 | 0.26527 |
| OSNR2 | 0.0892 | 0.23969 | 0.10547 | 0.12514 | 0.27248 | 0.38824 |
| OSNR3 | 0.09661 | 0.14491 | 0.13869 | 0.11392 | 0.3506 | 0.49597 |
| OSNR4 | 0.08157 | 0.15243 | 0.09365 | 0.19402 | 0.34398 | 0.4185 |
| OSNR5 | 0.05647 | 0.11818 | 0.29561 | 0.17464 | 0.26555 | 0.51054 |
| OSNR6 | 0.0667 | 0.12234 | 0.13169 | 0.17392 | 0.30204 | 0.66312 |
| OSNR7 | 0.06404 | 0.10733 | 0.11657 | 0.16572 | 0.2555 | 0.6632 |
| OSNR8 | 0.11962 | 0.09674 | 0.11411 | 0.13439 | 0.28545 | 0.7872 |
| OSNR9 | 0.09181 | 0.08717 | 0.08136 | 0.19993 | 0.30251 | 1.00502 |
| OSNR10 | 0.10112 | 0.10038 | 0.07975 | 0.18052 | 0.32175 | 1.08168 |
| OSNR11 | 0.09109 | 0.09711 | 0.08971 | 0.13505 | 0.3182 | 1.11133 |
| OSNR12 | 0.09903 | 0.13492 | 0.10886 | 0.20067 | 0.27411 | 1.01896 |
| OSNR13 | 0.10173 | 0.05981 | 0.08372 | 0.22835 | 0.41103 | 0.51775 |
| OSNR14 | 0.13015 | 0.07959 | 0.1037 | 0.37265 | 0.52585 | 1.04887 |
| OSNR15 | 0.09653 | 0.30351 | 0.11064 | 0.30835 | 0.44 | 1.43216 |
| OSNR16 | 0.10547 | 0.15373 | 0.11695 | 0.38168 | 0.47626 | 1.81007 |

**Figure 9 Original data set**

| Model | Image resolution: 64×64 | | Image resolution: 32×32 | |
| --- | --- | --- | --- | --- |
|  | Parameter Memory | FLOPs | Parameter Memory | FLOPs |
| ResNet18 | 42.63 | 296.099 | 42.63 | 74.03 |
| SA-ResNet | 2.605 | 161.876 | 2.605 | 40.47 |
| GoogLeNet | 22.815 | 258.243 | 22.815 | 64.571 |
| MobileNet-v2 | 8.453 | 48.915 | 8.453 | 12.242 |
| EfficientNet-b0 | 15.242 | 63.951 | 15.242 | 16.942 |

**Figure 10 Original data set**

| Model | Parameter memory | FLOPs |
| --- | --- | --- |
| Original network | 2.605 | 40.47 |
| 40% compression network | 1.574 | 30.499 |
| 60% compression network | 1.048 | 21.33 |
| 80% compression network | 0.517 | 9.471 |

**Figure 11 Original data set**

| Model | Accuracy，Sample length=20000 | | Accuracy，Sample length=16000 | |
| --- | --- | --- | --- | --- |
| Original network | 1 | | 1 | |
|  | Taylor pruning | Projection | Taylor pruning | Projection |
| 40% compression network | 1 | 1 | 1 | 1 |
| 60% compression network | 1 | 1 | 1 | 1 |
| 80% compression network | 1 | 0.99954 | 1 | 1 |

**Figure 12 Original data set**

| Model | Mean absolute error，Sample length=20000 | | Mean absolute error，Sample length=16000 | |
| --- | --- | --- | --- | --- |
| Original network | 0.35 | | 0.34 | |
|  | Taylor pruning | Projection | Taylor pruning | Projection |
| 40% compression network | 0.3 | 0.33 | 0.32 | 0.33 |
| 60% compression network | 0.32 | 0.42 | 0.32 | 0.38 |
| 80% compression network | 0.29 | 0.47 | 0.32 | 0.51 |

**Figure 13 Original data set**

| Physical parameters | Sample length | mean | standard deviation | mean | standard deviation | mean | standard deviation | mean | standard deviation | mean | standard deviation | mean | standard deviation | mean | standard deviation |
| --- | --- | --- | --- | --- | --- | --- | --- | --- | --- | --- | --- | --- | --- | --- | --- |
|  |  | Original network | | Taylor pruning, compression ratio 40% | | Projection, compression ratio 40% | | Taylor pruning, compression ratio 60% | | Projection, compression ratio 60% | | Taylor pruning, compression ratio 80% | | Projection, compression ratio 80% | |
| Different distances | | | | | | | | | | | | | | | |
| 160km | 20000 | 0.9999 | 2.33E-04 | 0.99998 | 3.88E-05 | 0.9999 | 2.33E-04 | 1 | 0 | 1 | 0 | 0.99991 | 1.94E-04 | 0.99986 | 2.18E-04 |
| 320km |  | 0.9999 | 2.33E-04 | 1 | 0 | 1 | 0 | 1 | 0 | 0.9999 | 2.33E-04 | 1 | 0 | 0.99979 | 2.50E-04 |
| 480km |  | 1 | 0 | 1 | 0 | 1 | 0 | 1 | 0 | 1 | 0 | 1 | 0 | 0.99995 | 1.16E-04 |
| 160km | 16000 | 0.99988 | 1.90E-04 | 0.9999 | 2.33E-04 | 0.9999 | 2.33E-04 | 0.99998 | 3.88E-05 | 0.99991 | 1.94E-04 | 0.99991 | 1.94E-04 | 0.99979 | 2.85E-04 |
| 320km |  | 0.99967 | 3.15E-04 | 0.99991 | 1.94E-04 | 0.9999 | 2.33E-04 | 0.99979 | 4.66E-04 | 0.99979 | 4.66E-04 | 0.99981 | 2.63E-04 | 0.99969 | 4.66E-04 |
| 480km |  | 0.99988 | 2.26E-04 | 1 | 0 | 1 | 0 | 1 | 0 | 1 | 0 | 1 | 0 | 1 | 0.00E+00 |
| Different phase deviation | | | | | | | | | | | | | | | |
| 0° | 20000 | 0.99995 | 1.16E-04 | 0.99998 | 3.88E-05 | 0.99979 | 4.66E-04 | 1 | 0.00E+00 | 0.99997 | 7.76E-05 | 1 | 0.00E+00 | 0.99983 | 1.74E-04 |
| 5° |  | 0.9999 | 2.33E-04 | 0.99998 | 3.88E-05 | 0.9999 | 2.33E-04 | 1 | 0.00E+00 | 1 | 0.00E+00 | 0.99991 | 1.94E-04 | 0.99986 | 2.18E-04 |
| 10° |  | 0.99969 | 4.66E-04 | 0.99981 | 2.77E-04 | 0.99977 | 4.58E-04 | 0.99988 | 2.26E-04 | 0.99979 | 3.11E-04 | 0.99977 | 3.28E-04 | 0.9996 | 2.18E-04 |
| 0° | 16000 | 0.9999 | 2.33E-04 | 1 | 0.00E+00 | 0.99979 | 4.66E-04 | 0.99998 | 3.88E-05 | 1 | 0.00E+00 | 0.9999 | 2.33E-04 | 0.99988 | 2.26E-04 |
| 5° |  | 0.99988 | 1.90E-04 | 0.9999 | 2.33E-04 | 0.9999 | 2.33E-04 | 0.99998 | 3.88E-05 | 0.99991 | 1.94E-04 | 0.99991 | 1.94E-04 | 0.99979 | 2.85E-04 |
| 10° |  | 0.99976 | 2.63E-04 | 0.99981 | 2.63E-04 | 0.99977 | 2.42E-04 | 0.99981 | 2.33E-04 | 0.99969 | 2.65E-04 | 0.99983 | 3.88E-04 | 0.99958 | 4.89E-04 |
| Different frequency offset | | | | | | | | | | | | | | | |
| 10MHz | 20000 | 0.9999 | 2.33E-04 | 0.99998 | 4.02E-05 | 0.9999 | 2.33E-04 | 1 | 0.00E+00 | 1 | 0.00E+00 | 0.99991 | 1.94E-04 | 0.99986 | 2.18E-04 |
| 100MHz |  | 0.99993 | 1.55E-04 | 1 | 0.00E+00 | 1 | 0.00E+00 | 1 | 0.00E+00 | 0.99998 | 3.88E-05 | 0.9999 | 2.33E-04 | 0.99972 | 2.63E-04 |
| 1000MHz |  | 0.99995 | 1.16E-04 | 1 | 0.00E+00 | 1 | 0.00E+00 | 1 | 0.00E+00 | 0.9999 | 2.33E-04 | 1 | 0.00E+00 | 0.99979 | 4.66E-04 |
| 10MHz | 16000 | 0.99988 | 1.90E-04 | 0.9999 | 2.33E-04 | 0.9999 | 2.33E-04 | 0.99998 | 3.88E-05 | 0.99991 | 1.94E-04 | 0.99991 | 1.94E-04 | 0.99979 | 2.85E-04 |
| 100MHz |  | 0.99969 | 2.85E-04 | 0.99979 | 4.66E-04 | 0.99979 | 2.85E-04 | 0.99979 | 4.66E-04 | 0.99977 | 3.11E-04 | 0.99979 | 4.66E-04 | 0.99983 | 2.68E-04 |
| 1000MHz |  | 0.9999 | 2.33E-04 | 0.99997 | 7.76E-05 | 0.99988 | 1.69E-04 | 1 | 0.00E+00 | 0.99997 | 4.75E-05 | 1 | 0.00E+00 | 0.99988 | 1.70E-04 |

**Figure 14 Original data set**

| Physical parameters | Sample length | mean | standard deviation | mean | standard deviation | mean | standard deviation | mean | standard deviation | mean | standard deviation | mean | standard deviation | mean | standard deviation |
| --- | --- | --- | --- | --- | --- | --- | --- | --- | --- | --- | --- | --- | --- | --- | --- |
|  |  | Original network | | Taylor pruning, compression ratio 40% | | Projection, compression ratio 40% | | Taylor pruning, compression ratio 60% | | Projection, compression ratio 60% | | Taylor pruning, compression ratio 80% | | Projection, compression ratio 80% | |
| Different distances | | | | | | | | | | | | | | | |
| 160km | 20000 | 0.30334 | 2.05E-02 | 0.27794 | 1.88E-02 | 0.31389 | 2.02E-02 | 0.29818 | 9.86E-03 | 0.34264 | 1.78E-02 | 0.30233 | 2.34E-02 | 0.42901 | 1.90E-02 |
| 320km |  | 0.33826 | 1.18E-02 | 0.34616 | 4.48E-03 | 0.33893 | 1.87E-02 | 0.33507 | 2.66E-02 | 0.38634 | 2.20E-02 | 0.35905 | 1.66E-02 | 0.49988 | 2.41E-02 |
| 480km |  | 0.39645 | 8.72E-03 | 0.39995 | 7.03E-03 | 0.37663 | 9.16E-03 | 0.39168 | 3.11E-02 | 0.44888 | 2.53E-02 | 0.40797 | 1.64E-02 | 0.53322 | 2.57E-02 |
| 160km | 16000 | 0.3158 | 1.81E-02 | 0.29231 | 1.82E-02 | 0.32639 | 2.33E-02 | 0.29926 | 1.23E-02 | 0.37227 | 2.15E-02 | 0.30289 | 2.86E-03 | 0.47069 | 8.10E-03 |
| 320km |  | 0.37695 | 3.00E-02 | 0.36215 | 1.27E-02 | 0.35388 | 9.31E-03 | 0.35231 | 8.78E-03 | 0.40697 | 2.06E-02 | 0.37604 | 3.01E-02 | 0.52085 | 2.38E-02 |
| 480km |  | 0.40947 | 3.29E-02 | 0.40383 | 1.47E-02 | 0.4138 | 8.08E-03 | 0.41268 | 1.87E-02 | 0.44641 | 1.97E-02 | 0.41851 | 1.91E-02 | 0.57626 | 3.70E-02 |
| Different phase deviation | | | | | | | | | | | | | | | |
| 0° | 20000 | 0.25211 | 1.03E-02 | 0.25757 | 1.35E-02 | 0.25109 | 9.74E-03 | 0.25806 | 1.98E-02 | 0.28087 | 1.96E-02 | 0.26933 | 1.05E-02 | 0.37233 | 1.53E-02 |
| 5° |  | 0.30334 | 2.05E-02 | 0.27794 | 1.88E-02 | 0.31389 | 2.02E-02 | 0.29818 | 9.86E-03 | 0.34264 | 1.78E-02 | 0.30233 | 2.34E-02 | 0.42901 | 1.90E-02 |
| 10° |  | 0.42096 | 1.47E-02 | 0.43309 | 9.91E-03 | 0.43967 | 2.92E-02 | 0.42079 | 1.82E-02 | 0.49675 | 2.32E-02 | 0.44998 | 2.23E-02 | 0.60053 | 2.91E-02 |
| 0° | 16000 | 0.27568 | 3.85E-03 | 0.28885 | 9.46E-03 | 0.26414 | 1.74E-02 | 0.2766 | 1.52E-02 | 0.30902 | 1.60E-02 | 0.28844 | 5.79E-03 | 0.39727 | 1.74E-02 |
| 5° |  | 0.3158 | 1.81E-02 | 0.29231 | 1.82E-02 | 0.32639 | 2.33E-02 | 0.29926 | 1.23E-02 | 0.37227 | 2.15E-02 | 0.30289 | 2.86E-03 | 0.47069 | 8.10E-03 |
| 10° |  | 0.44874 | 3.21E-02 | 0.46407 | 1.67E-02 | 0.46071 | 2.14E-02 | 0.45068 | 3.11E-02 | 0.50267 | 2.77E-02 | 0.47092 | 2.05E-02 | 0.65162 | 2.03E-02 |
| Different frequency offset | | | | | | | | | | | | | | | |
| 10MHz | 20000 | 0.30334 | 2.05E-02 | 0.27794 | 1.88E-02 | 0.31389 | 2.02E-02 | 0.29818 | 9.86E-03 | 0.34264 | 1.78E-02 | 0.30233 | 2.34E-02 | 0.42901 | 1.90E-02 |
| 100MHz |  | 0.30178 | 2.29E-02 | 0.30593 | 1.84E-02 | 0.2957 | 1.57E-02 | 0.29957 | 1.26E-02 | 0.35146 | 1.84E-02 | 0.31479 | 1.54E-02 | 0.43195 | 1.81E-02 |
| 1000MHz |  | 0.3207 | 1.09E-02 | 0.32328 | 1.78E-02 | 0.31738 | 1.12E-02 | 0.32601 | 2.54E-02 | 0.35168 | 1.17E-02 | 0.3391 | 2.94E-02 | 0.45747 | 1.62E-02 |
| 10MHz | 16000 | 0.3158 | 1.81E-02 | 0.29231 | 1.82E-02 | 0.31389 | 2.02E-02 | 0.29926 | 1.23E-02 | 0.32639 | 2.33E-02 | 0.30289 | 2.86E-03 | 0.47069 | 8.10E-03 |
| 100MHz |  | 0.33769 | 1.56E-02 | 0.33599 | 5.69E-03 | 0.33889 | 2.06E-02 | 0.34816 | 1.06E-02 | 0.37635 | 2.51E-02 | 0.34488 | 2.91E-02 | 0.49082 | 1.29E-02 |
| 1000MHz |  | 0.3379 | 1.38E-02 | 0.3373 | 2.73E-02 | 0.3413 | 1.84E-02 | 0.32896 | 1.24E-02 | 0.37997 | 1.27E-02 | 0.35448 | 1.59E-02 | 0.49668 | 1.78E-02 |
